# Corneal Epithelium Thickness Profile in 614 Normal Chinese Children Aged 7–15 Years Old

**DOI:** 10.1038/srep23482

**Published:** 2016-03-23

**Authors:** Yingyan Ma, Xiangui He, Xiaofeng Zhu, Lina Lu, Jianfeng Zhu, Haidong Zou

**Affiliations:** 1 Shanghai Eye Hospital, Shanghai Eye Disease Prevention & Treatment Center, No. 380 KangDing Road, Shanghai 200040, China; 2 Department of Ophthalmology, Shanghai General Hospital, Shanghai Jiao Tong University, School of Medicine, No. 100 HaiNing Road, Shanghai 200080, China; 3 Department of Maternal and Child Health, School of Public Health, Key Laboratory of Public Health Safety, Ministry of Education, Fudan University, No. 130 DongAn Road, Shanghai 200032, China

## Abstract

The purpose of the study is to describe the values and distribution of corneal epithelium thickness (CET) in normal Chinese school-aged children, and to explore associated factors with CET. CET maps were measured by Fourier-domain optical coherence tomography (FD-OCT) in normal Chinese children aged 7 to 15 years old from two randomly selected schools in Shanghai, China. Children with normal intraocular pressure were further examined for cycloplegic autorefraction, corneal curvature radius (CCR) and axial length. Central (2-mm diameter area), para-central (2- to 5-mm diameter area), and peripheral (5- to 6-mm diameter area) CET in the superior, superotemporal, temporal, inferotemporal, inferior, inferonasal, nasal, superonasal cornea; minimum, maximum, range, and standard deviation of CET within the 5-mm diameter area were recorded. The CET was thinner in the superior than in the inferior and was thinner in the temporal than in the nasal. The maximum CET was located in the inferior zone, and the minimum CET was in the superior zone. A thicker central CET was associated with male gender (p = 0.009) and older age (p = 0.037) but not with CCR (p = 0.061), axial length (p = 0.253), or refraction (p = 0.351) in the multiple regression analyses. CCR, age, and gender were correlated with para-central and peripheral CET.

Measuring profiles of corneal epithelium thickness (CET) in normal population could provide references to define any abnormal thinning or thickening, or change of the distribution pattern in some disease states. Keratoconus usually initiates during adolescence and slowly progresses until the age of 30 or 40 years old^1^. However, compared with in adults, keratoconus was significantly more severe at diagnosis in children^2^. In addition, progression of keratoconus can be markedly faster in children, leading to amblyopia and visual impairment, and is one of the most common reasons for corneal transplantation in children^3^. With the development of effective therapeutic options, diagnostic methods and their corresponding criteria to detect keratoconus at early stages could be of great importance. Several studies reported the diagnosis of keratoconus by measuring CET maps. Keratoconic eyes had thinner center and minimum CET and varied more widely in the CET maps than normal eyes^4^,^5^,^6^,^7^,^8^,^9^. In a recent study, diagnosis of keratoconus was classified into 5 stages by changes of the corneal structures, in which thinning of the epithelium pachymetry at the conus is an important manifestation in the earliest stage^10^. Profiles of CET maps in normal children could provide valuable references for estimating abnormal values or distribution of the CET, which might be an adjunctive indicator for early keratoconus.

In addition, school-aged children were at high prevalence of suffering from various refractive errors, which could usually be corrected with spectacles or contact lenses. Wearing orthokeratology (OK) contact lenses could correct visual acuity by epithelium remodeling and was reported to effectively retard progression of myopia with its special control of the relative peripheral hyperopia^11^. Measuring profiles of CET maps is also valuable for assessing the safety of corneal epithelium remodeling during the treatment, exploring possible mechanisms behind, or designing the contact lenses^12^. However, most of the existing literature about normal CET values and distributions was performed in adult populations; few studies measured CET maps in normal children. Qian and associates included 44 normal children at a mean age of 11.1 ± 1.9 years in the control group of their study, which aimed to explore the characteristics of CET maps in children wearing myopic OK contact lenses^12^. However, the sample size was too small to infer reference ranges for normal children.

Several methods have been introduced to measure CET, such as confocal microscopy^13^,^14^, optical pachymetry^15^, optical coherence tomography (OCT)^16^,^17^, high frequency ultrasound biomicroscopy^8^, and very high frequency digital ultrasound^18^. Fourier-domain optical coherence tomography (FD-OCT) is a high-speed, high-resolution, non-contact, 3-dimensional OCT, which has been applied in measuring CET for patients with keratoconus, corneal ectasia and dry eye, patients undergoing LASIK surgery, patients wearing soft or hard contact lenses and normal patients with relatively high repeatability^6^,^12^,^19^,^20^,^21^,^22^,^23^,^24^. This study presented CET maps of the central (2-mm diameter area), para-central (2- to 5-mm diameter area) and peripheral (6-mm diameter area) corneas in normal Chinese school children using FD-OCT. The values and the positions of the minimum and the maximum CET were also investigated. Regression analyses were performed to explore possible associated factors for central, para-central, and peripheral CET.

## Results

A total of 648 schoolchildren participated in the study, and CET of 1228 eyes from 614 children was included in the analyses. Reasons for exclusion were recent wearing of soft or hard contact lenses (6 subjects), history of ocular diseases except for refractive error (2 subjects), and uncooperative with examinations or lack of complete CET data from both two eyes (26 subjects). Among those included in the analyses, 312 (50.8%) were male. The mean age was 11.24 (Standard deviation (SD) = 2.34).

**Figure 1:**
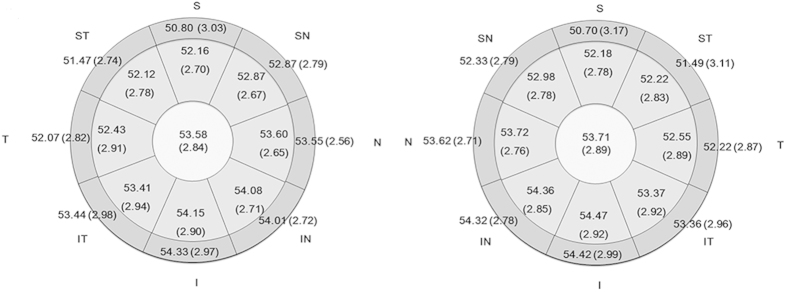
Pachymetry map for corneal epithelium thickness measured by RTVue Fourier optical coherence tomography for 614 Chinese children in right (Left) and left eyes (Right) (Mean (standard deviation), μm). S: Superior, ST: Superior Temporal, ST: Temporal, IT: Inferior Temporal, I: Inferior, IN: Inferior Nasal, N: Nasal, SN: Superior Nasal.

**Figure 2:**
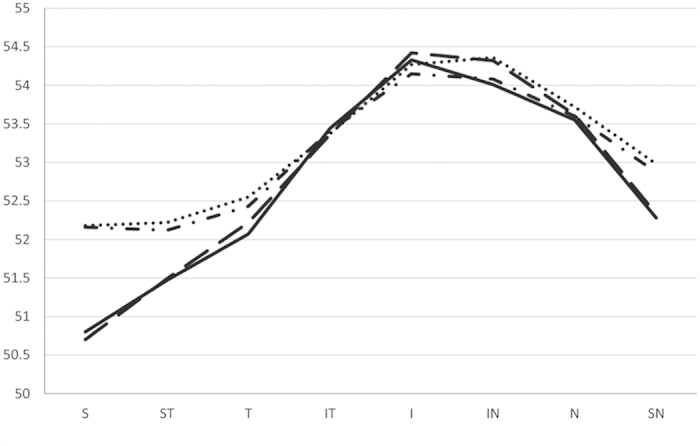
Para-central (2- to 5-mm diameter) and peripheral (5- to 6-mm diameter) corneal epithelium thickness in the right and left eyes of 614 normal Chinese children (Mean (standard deviation), μm). Dot dash line: CET from the right eyes in the 2- to 5-mm diameter corneal; dotted line: CET from the left eyes in the 2- to 5-mm diameter regions; full line: CET from the right eyes in the 5- to 6-mm diameter corneal; dashed line: CET from the left eyes in the 5- to 6-mm diameter cornea. CET: corneal epithelium thickness, S: Superior, ST: Superior Temporal, ST: Temporal, IT: Inferior Temporal, I: Inferior, IN: Inferior Nasal, N: Nasal, SN: Superior Nasal.

Table 1 summarizes the CET in the central and peripheral areas from both eyes. CET did not follow a normal distribution (for example, central CET in the right eye, Kolmogorov-Smirnov Z test, p < 0.001). The CET was highly correlated between the right eye and left eye (all P < 0.01) (Table 1). The values of CET in the para-central cornea (2- to 5-mm diameter) were close to that in the peripheral cornea (5- to 6-mm diameter). The largest difference between the para-central and peripheral CET was in the superior part of the cornea (Figs 1 and 2). Within the 5-mm diameter area, the average CET in the superior half of the corneas was 52.48 ± 2.64 μm in right eyes, and 52.58 ± 2.71 μm in left eyes, thinner than the average CET in the inferior half of the corneas (53.69 ± 2.74 μm in right eyes, 53.80 ± 2.81 μm in left eyes, p < 0.001 for both the right and left eyes).

In the area 5 mm from the pupil center, the minimum epithelium thickness was located in the superior section of the cornea, followed by the superior temporal section in both right and left eyes. The maximum CET was located in the inferior section of the cornea, followed by the inferior nasal section in both right and left eyes. The average values for the minimum and maximum CET, their differences, and the standard deviation of CET in the 5-mm diameter area are given in Table 2.

To explore possible correlated factors with the CET, 398 children with normal intraocular pressure and with written informed consent for cycloplegia participated further examinations. Excluding children who are uncooperative with examinations and those without complete data, 370 right eyes from 370 children with complete measurements of cycloplegic autorefraction, axial length, corneal radius, and intraocular pressure were included. The mean age was 11.61 (SD = 2.13), and 168 (45.41%) were boys. The age was statistically older (student-t test, P < 0.001), and the proportion of male gender was significantly less (chi-square test, P = 0.001) in children who were included in the further analyses than those were not included, probably because older children and girls are more cooperative with examinations than younger children and boys.

Central CET (CCET) (2-mm diameter area) was associated with gender (p = 0.009) and age (p = 0.037). Boys had thicker epithelium thickness than girls; older children had thicker CET than younger children. Corneal curvature radius (CCR) (p = 0.061), axial length (p = 0.253), and refraction (p = 0.351) were not associated with CCET (Table 3). However, in the para-central and peripheral cornea, CCR was correlated with CET in all zones. Age was positively associated with para-central and peripheral CET in most of the zones, except in the superior (2 to 5 mm) and superior nasal (5 to 6 mm) regions. Males had thicker para-central and peripheral CET, except in both the 2- to 5-mm and 5- to 6-mm diameter regions in the inferior temporal, inferior, and inferior nasal parts and the superior nasal (5 to 6 mm) part (Table 3). The mean CET values according to age interval, gender, and quartile CCR were calculated in Table 4, 5, and 6.

## Discussion

This is the first study describing the values, distribution pattern, and correlated factors for the central (2-mm diameter area), para-central (2- to 5-mm diameter area), and peripheral (5- to 6-mm diameter area) CET in children with a large sample size. In the para-central and peripheral cornea, the CET was thinner in the superior than in the inferior and in the temporal than in the nasal. The maximum CET was located in the inferior zone, and the minimum CET was located in the superior zone in the 2- to 5-mm diameter annulus. Moreover, the CETs were associated with gender, age, and CCR according to the multiple regression analyses.

The CET values could be partially affected by the instruments used for measurement. Confocal microscopy or confocal microscopy through focusing measured relatively lower CET values than spectral domain optical coherence tomography or a very high-frequency digital ultrasound arc-scanner^13^,^14^. Compared with the CCET measured by FD-OCT, our results were between the largest value of 58.6 ± 4.6 μm in 120 healthy subjects of 24.2 ± 2.6 year olds and the smallest value of 48.3 ± 3.0 μm in 18 normal subjects of 28.5 ± 6.5 year olds^23^,^25^. Compared with the CCET of 52.8 ± 2.0 μm in 44 normal children 11.1 ± 1.9 years old reported in Qian and associates’ study, our results were a litter thicker^12^. However, with relatively large sample size, which meets the requirements of statistics, the present study provides more accurate and reliable values of CET. The distribution pattern described in the present study was consistent with Yang and associates’ study measured by FD-OCT in a group of people with an average age of about 40 years and Reinstein and associates’ study using a very high frequency digital ultrasound scanner in people older than 20 years old, both of which were within the 6-mm diameter area^18^,^26^.

Only a few studies reported the CET and associated factors. Tao and associates’ did not observe a significant association between the CET and gender or age in 44 eyes from 22 people^27^. Francoz and associates found no difference of CET in normal adults younger than 40 years old (n = 18) and adults older than 40 years old (n = 10)^23^. Reinstein and associates reported a non-significant relationship between vertex CET and age, refraction, and keratometry in 56 subjects^18^. However, the sample sizes were too small to infer any possible relationships. In a recent study that included 97 normal eyes, males had thicker CET than females, similar to our results^28^. It is plausible that males have thicker CET than females because the total corneal thickness was reported to be thicker in males than in females^29^,^30^. As one layer of the total corneal thickness, CET could also bear a gender difference, as observed in the total corneal thickness^29^,^30^. Hormone levels may be involved in the corneal thickness and topography^31^.

Total ocular refraction consists of corneal refraction, axial length and lens refraction, which is why CET was not found to be associated with spherical equivalent refraction. However, in the present study, thicker para-central and peripheral CETs were positively associated with larger CCR (ambiguously for central CET, p = 0.061). The relationship between CCR and CET has rarely been reported. Previous studies only identified the relationship between the total corneal thickness and CCR. A thicker total corneal thickness was correlated with a larger CCR, and a flatter cornea was correlated with smaller corneal refraction^29^,^32^. In the present study, as the outermost layer of cornea, we inferred that the corneal epithelium contributes to corneal refraction, probably through its thickness, which is in accordance with the mechanisms of RGP or OK contact lenses to correct refractive error, in which CETs are thickened or thinned in the center and in the peripheral cornea according to different design, leading to a change in corneal refraction^11^,^33^,^34^. The non-uniformity of the CET observed in normal children could also explain the possible mechanism of the distribution pattern of CET after wearing OK contact lenses. It was reported that whereas the mid-peripheral nasal and temporal CET thickened after overnight wearing of OK contact lenses, the mid-peripheral superior CET thinned and the inferior CET did not change significantly^35^. Hence, when designing OK contact lenses for children, the normal distribution pattern of CET can be considered to make the contact lenses better fitted to their corneas, avoiding decentration of the lenses. In addition, because infectious keratitis can occur through the thinning epithelium^36^, measuring the epithelium thickness might be helpful to decrease possible adverse events of wearing OK contact lenses. Therefore, it is suggested that measuring CET maps could be included as a routine examination when prescribing OK contact lenses for children.

An association between age and CET was also observed in the present study. Children experienced a growth of CET in the center, para-center, and periphery with age. Total corneal thickness was reported to increase with age in children until 11 years old^37^; however the growth pattern of CET in children is not addressed in previous literatures. Yang and associates demonstrated that age was inversely correlated with CET in 180 normal adults^26^. However, the number of children included in their study was relatively small. Combined with their results, it is possible to infer that CET might undergo an increase during childhood and then a decrease after entering adulthood. A brief review of the variation parameters in normal people by FD-OCT showed that compared with studies in adults with a mean age over 40 years old, the minimum CET was thicker. The SD and the absolute difference of MIN-MAX were smaller in school-aged children in the present study (Supplementary information files: Supplementary Table 1). Hence, age differences in CET should be considered when establishing threshold values for the prognosis of keratoconus when applying those parameters in clinical practice in the future. However, the maximum CET was stable among different age groups, which coincided with Yang and associates’ findings^26^. Future study could analyze the value of the maximum CET in the prognosis of keratoconus because this parameter is stable among difference age groups.

In addition to age-related influences on CET, the CET distribution pattern in normal children observed in the present study could also be a reference in estimating abnormal distribution of CET in some disease states. In patients with keratoconus, the normal distribution pattern is altered, especially in the horizontal meridian^4^,^9^. Reinstein and associates found an epithelial doughnut pattern manifested by keratoconic eyes, with a thinning of CET at keratoconic protrusion surrounded by an annulus of CET thickening, which was confirmed in a recent study defining this phenomenon in the first stage of keratoconus^5^,^10^. Keratotic protrusions are most frequently presented in the inferotemporal cornea, accordingly, the CET in keratotic eyes were reported to be thinner inferotemporally and thicker supranasally compared with in normal eyes, resulting in an inverse distribution pattern along the horizontal meridian^4^,^9^. Moreover, the minimum CET in the form fruste keratoconus was located in the inferior parts of the cornea instead of the superior parts which was observed in normal eyes^7^. Since the epithelium could compensate change of stromal structure in keratonus, changes of the epithelium could precede changes on the front surface of the cornea, making the front surface topography within the normal range^5^. Hence, the distribution pattern of the CET, as well as the values, could be evaluated adjunctively with corneal topography and other diagnostic methods in the early prognosis of keratoconus, however its true value in this regard remains to be determined. With the development of effective treatment methods, such as corneal collagen cross-linking, early prognosis of keratoconus could be of great benefit^38^.

Like most studies, the present study has some limitations. First, only two schools in Shanghai were included, which might influence the generalization of the study. The sample size was relatively small when the means for each age interval were determined; however, the study was qualified according to statistics^39^. Second, the CET measured by FD-OCT included the thickness of tear film of approximately 3 μm^40^, which might overestimate the true values for CET and influence the possible correlated factors of CET. However, the distribution pattern reported in the present study was similar to what was reported using very high frequency ultrasound, which excluded the thickness of tear film^18^. An instrument with even higher resolution to identify and exclude tear film thickness is needed to produce more accurate measurements for CET. Third, the CET profiles presented in our study covered 6-mm diameter regions of the cornea, which might not be enough for the diagnosis of peripheral cornea diseases. For the diagnosis of corneal diseases that infringe upon more peripheral parts of the cornea, such as marginal degeneration, thickness profiles that cover a larger area are needed. However, a 6-mm diameter might be sufficient for keratoconus^4^.

Strengths of the study include the high repeatability of FD-OCT in measuring CET, with five times repeated in 1.55 seconds. In addition, the total number of the sample size is relatively large to evaluate the CET values and distribution in school-aged children. Despite the school-based design of the study, the present study population could be representative, since the attendance rate of primary schools and middle schools for children aged 7 to 15 years old in Shanghai is 99.9%. Moreover, the personnel conducted the examinations had been uniformly trained and tested before the examinations to ensure the quality of the study.

In conclusion, the present study provided CET maps for children aged 7 to 15 years by FD-OCT, showing the distribution pattern of CE and the factors correlated with CET in the center (2-mm diameter), para-center (2- to 5-mm diameter), and periphery (5- to 6-mm diameter) cornea. CET experiences mild growth in school-aged children. In addition, male gender and a flatter cornea were associated with a thicker CET.

## Methods

Two schools, one primary school and one secondary school, from Shanghai, China were randomly selected by the cluster sampling method. Students with odd code numbers were included in the study. Inclusion criteria were children of Chinese nationality, aged 7 to 15 years old. Exclusion criteria were children with ocular diseases except for refractive error, or children with a history of wearing soft or hard contact lenses (in the recent 4 weeks). Children who were uncooperative with the examination or without written informed consent from their parents or guardians were also excluded. To obtain a 95% confidence interval where the mean falls within the range of one third of the expected standard deviation of 3 μm, the sample size required is 35 for each age interval^26^,^39^.

The study adhered to the tenets of the Declaration of Helsinki and was approved by the Institutional Review Board at the Shanghai General Hospital, Shanghai Jiao Tong University. Written informed consents were obtained from the guardians of all the children, and from both children and their guardians if the participants were 10 years or older. Oral agreement was also obtained by children if they were six years or older. Children and parents could choose whether to accept cycloplegia or not. The examinations were conducted during weekdays when schools were in session in May and June, 2013. One ophthalmologist, three optometrists, and two public health doctors participated in conducting the examinations. Children first underwent a basic eye examination including uncorrected visual acuity (Standard Logarithmic Visual Acuity E Chart, 5 m) and slit lamp examination.

CET measurements were then performed by RTVue FD-OCT (Optovue, Inc., Fremont, CA, USA) with a wide-angle (long) corneal adapter module lens (CAM) in the “pachymetry” scan pattern (6-mm diameter scan, 8 meridians, 1024 axial scans each, repeated 5 times). The children’s heads were stabilized with a chin rest, and they were asked to stare at an internal fixation target in the OCT. The pachymetry scans were centered on the pupil, and the images were displayed on screen in real time to help with alignment. If the children rotated the eyes or blinked during the measurement, another scan was taken to ensure the quality. To measure the test-retest repeatability of the FD-OCT for CET, the first 25 students from each school were selected. The intraclass correlation coefficient values between the two measurement were 0.941 for the central CET. RTVue-CAM software (version 6.11) automatically processed the OCT scan to provide the pachymetry map for CET (within the 6-mm diameter range), including the values and locations of the minimum and maximum thickness, the average values of the superior and inferior CET, the standard deviation, and the differences between the maximum and the minimum CET within the central 5-mm diameter region. The maps were divided into 17 zones: one 2-mm diameter pupil center (C), eight 2- to 5-mm para-central sectors (superior (S), superotemporal (ST), temporal (T), inferotemporal (IT), inferior (I), inferonasal (IN), nasal (N), superonasal (SN)) and eight 5- to 6-mm diameter peripheral sectors (S, ST, T, IT, I, IN, N, SN)(Fig. 1). The average thickness of each zone was calculated and displayed automatically in the corresponding region for CET (Fig. 1).

After CET measurement, the intraocular pressure (IOP) was recorded by noncontact tonometer (T-1000, Nidek, Japan), and children with normal IOP who had written informed consent from their parents to undergo cycloplegia were given cycloplegia by 1% cyclopentolate (Cyclogyl; Alcon, Fort Worth, TX, USA), followed by autorefraction, corneal curvature radius by an autorefractor (KR-8800, Topcon, Tokyo), axial length by IOLMaster (version 5.02, Carl Zeiss Meditec, Oberkochen, Germany), and best-corrected visual acuity if one had uncorrected visual acuity lower than 20/25 in either eye.

A one-sample Kolmogorov-Smirnov Z test was used to evaluate the normalcy of distribution of the CET. The Wilcoxon signed rank test was used to compare the differences of CET between the right and left eyes, and the Spearman correlation coefficient was used to evaluate the correlations between the right and left eyes. Multiple linear regressions were performed to explore the effects of sex, age, axial length, refraction (spherical equivalent = spherical degree + 0.5* cylindrical degree), and corneal curvature radius on CET. P values of less than 0.05 were considered statistically significant. SPSS 16.0 (SPSS Institute, Inc., Chicago, IL) was used for the statistical analyses.

## Additional Information

**How to cite this article**: Ma, Y. *et al*. Corneal Epithelium Thickness Profile in 614 Normal Chinese Children Aged 7–15 Years Old. *Sci. Rep.* **6**, 23482; doi: 10.1038/srep23482 (2016).

## Supplementary Material

Supplementary Information

## Figures and Tables

**Table 1:** Corneal Epithelium Thickness in the central (2-mm diameter), para-central (2 to 5- mm diameter), and peripheral (5 to 6-mm diameter) in right and left eyes of 614 normal Chinese children.

	Right Eye		Left Eye		Correlation Coefficient^a^	Wilcoxon Signed Rank Test^b^
	Mean (μm)	SD	Mean (μm)	SD		P value
Center	53.58	2.84	53.71	2.89	0.87	0.023
Superior (2–5 mm)	52.16	2.70	52.18	2.78	0.80	0.772
Superior (6 mm)	50.80	3.03	50.70	3.17	0.67	0.376
Superior Temporal (2–5 mm)	52.12	2.78	52.22	2.83	0.84	0.067
Superior Temporal (6 mm)	51.47	2.74	51.49	3.11	0.73	0.832
Temporal (2–5 mm)	52.43	2.91	52.55	2.89	0.84	0.096
Temporal (6 mm)	52.07	2.82	52.22	2.87	0.78	0.050
Inferior Temporal (2–5 mm)	53.41	2.94	53.37	2.92	0.85	0.261
Inferior Temporal (6 mm)	53.44	2.98	53.36	2.96	0.82	0.119
Inferior (2–5 mm)	54.15	2.90	54.27	2.92	0.85	0.045
Inferior (6 mm)	54.33	2.97	54.42	2.99	0.85	0.260
Inferior Nasal (2–5 mm)	54.08	2.71	54.36	2.85	0.85	<0.001
Inferior Nasal (6 mm)	54.01	2.72	54.32	2.78	0.84	<0.001
Nasal (2–5 mm)	53.60	2.65	53.72	2.76	0.85	0.021
Nasal (6 mm)	53.55	2.56	53.62	2.71	0.83	0.294
Superior Nasal (2–5 mm)	52.87	2.67	52.98	2.78	0.84	0.089
Superior Nasal (6 mm)	52.28	2.79	52.33	2.79	0.73	0.419

^a^Spearman’s Rank Correlation Coefficient was used to evaluate the correlations of corneal epithelium thickness in various zones between right and left eyes of 614 normal Chinese children. All P < 0.001.

^b^Wilcoxon Signed Rank Test was used to test the differences of corneal epithelium thickness in various zones between right and left eyes of 614 normal Chinese children.

**Table 2:** Values of the minimum, maximum corneal epithelium thickness, standard deviation, and the differences of minimum and maximum corneal epithelium thickness in the 5-mm diameter area in the right and left eyes of 614 Chinese children (Mean ± SD).

	Right eye (μm)	Left eye (μm)
Minimum CET	50.01 ± 2.84	49.90 ± 3.01
Maximum CET	56.03 ± 3.20	56.18 ± 3.14
Standard Deviation	1.37 ± 0.58	1.43 ± 0.58
Min–Max	−6.01 ± 2.72	−6.30 ± 2.76

CET, corneal epithelium thickness.

Min–Max, differences of minimum CET and maximum CET.

SD, standard deviation.

**Table 3:** Associated factors for the corneal epithelium thickness in the central (2-mm diameter), para-central (2 to 5-mm diameter), and peripheral (5 to 6-mm diameter) cornea by multiple regression analysis (n = 370, data from right eyes).

Dependent Variable	Independent Variables Included in Equation	Beta Coefficient	95% CI of Beta Coefficient	P Value
Center	Gender	−0.73	−1.28 to −0.18	0.009
	Age	0.14	0.01 to 0.27	0.037
Superior (2–5 mm)	CCR	1.86	0.84 to 2.88	<0.001
	Gender	−0.71	−1.25 to −0.17	0.010
Superior (6 mm)	CCR	2.60	1.52 to 3.69	<0.001
	Age	−0.14	−0.27 to −0.00	0.048
Superior Temporal (2–5 mm)	Gender	−1.03	−1.58 to −0.49	<0.001
	Age	0.22	0.10 to 0.34	0.001
	CCR	1.35	0.32 to 2.37	0.010
Superior Temporal (6mm)	Gender	−0.76	−1.31 to −0.22	0.006
	CCR	1.73	0.71 to 2.76	0.001
	Age	0.17	0.05to 0.29	0.007
Temporal (2–5 mm)	Age	0.29	0.16 to 0.42	<0.001
	Gender	−0.86	−1.43 to −0.30	0.003
	CCR	1.23	0.16 to 2.30	0.024
Temporal (6 mm)	Age	0.30	0.18 to 0.43	<0.001
	Gender	−0.85	−1.41 to −0.29	0.003
	CCR	1.53	0.47 to 2.58	0.005
Inferior Temporal (2–5 mm)	Age	0.30	0.17 to 0.43	<0.001
	CCR	1.69	0.62 to 2.75	0.002
Inferior Temporal (6 mm)	Age	0.33	0.19 to 0.47	<0.001
	CCR	1.92	0.81 to 3.02	0.001
Inferior (2–5 mm)	Age	0.25	0.12 to 0.39	<0.001
	CCR	1.52	0.44 to 2.60	0.006
Inferior (6 mm)	Age	0.29	0.15 to 0.42	<0.001
	CCR	1.95	0.85 to 3.06	0.001
Inferior Nasal (2–5 mm)	Age	0.25	0.12 to 0.37	<0.001
	CCR	1.95	0.92 to 2.97	<0.001
Inferior Nasal (6 mm)	CCR	2.15	1.11 to 3.19	<0.001
	Age	0.26	0.13 to 0.39	<0.001
Nasal (2–5 mm)	Age	0.25	0.12 to 0.37	<0.001
	CCR	1.56	0.54 to 2.56	0.003
	Gender	−0.69	−1.23 to −0.15	0.013
Nasal (6 mm)	CCR	1.93	0.94 to 2.92	<0.001
	Age	0.24	0.12 to 0.36	<0.001
	Gender	−0.70	−1.22 to −0.17	0.010
Superior Nasal (2–5 mm)	CCR	1.72	0.71 to 2.73	0.001
	Gender	−0.73	−1.27 to −0.20	0.008
	Age	0.12	0.00 to 0.24	0.050
Superior Nasal (6 mm)	CCR	2.77	1.74 to 3.79	<0.001

CCR: corneal curvature radius.

CI: confidence interval.

**Table 4:** Corneal epithelium thickness in the central (2-mm diameter), para-central (2 to 5-mm diameter), and peripheral (5 to 6-mm diameter), according to each age interval in 614 normal Chinese children.

	Age																			
	7		8		9		10		11		12		13		14		15		Total	
	Mean	SD	Mean	SD	Mean	SD	Mean	SD	Mean	SD	Mean	SD	Mean	SD	Mean	SD	Mean	SD	Mean	SD
	n = 54		n = 43		n = 63		n = 62		n = 86		n = 93		n = 88		n = 88		n = 37		n = 614	
C	53.24	2.84	52.33	2.38	53.38	2.86	53.74	2.36	53.22	2.69	53.68	3.07	54.01	2.61	53.76	2.98	54.78	3.38	53.58	2.84
S1	52.61	2.72	51.37	2.41	52.10	2.61	52.29	2.29	51.88	2.51	52.11	2.85	52.63	2.69	52.13	2.84	52.05	3.40	52.16	2.70
S2	51.74	2.83	50.79	3.17	51.40	2.69	51.48	2.49	50.65	2.33	50.55	3.17	50.82	3.25	50.14	3.43	49.73	3.52	50.80	3.03
ST1	52.00	2.69	50.70	2.39	51.81	2.99	51.94	2.35	51.76	2.64	52.13	2.76	52.90	2.75	52.53	2.79	52.76	3.37	52.12	2.78
ST2	51.39	3.34	50.67	2.39	51.25	3.10	51.45	2.36	51.27	2.46	51.49	2.67	52.17	2.70	51.63	2.67	51.35	3.08	51.47	2.74
T1	51.65	2.95	50.72	2.25	51.97	2.83	52.02	2.91	52.01	2.67	52.56	2.72	53.31	2.58	53.05	2.97	54.14	3.58	52.43	2.91
T2	51.15	2.82	50.44	2.21	51.41	2.68	51.55	2.92	51.87	2.64	52.24	2.55	52.93	2.54	52.70	2.83	53.73	3.42	52.07	2.82
IT1	52.61	3.18	51.63	2.28	52.83	2.95	53.26	2.57	52.99	2.63	53.54	2.91	54.13	2.51	54.05	3.14	55.30	3.41	53.41	2.94
IT2	52.54	3.62	51.56	2.27	52.60	2.97	53.27	2.31	53.33	2.62	53.72	3.03	54.14	2.59	54.05	3.15	55.11	3.14	53.44	2.98
I1	53.52	2.98	52.56	2.41	53.70	3.00	54.03	2.33	53.81	2.50	54.26	2.97	54.69	2.48	54.69	3.22	55.81	3.49	54.15	2.90
I2	53.65	2.93	52.79	2.59	53.62	3.07	54.16	2.48	54.19	2.65	54.58	3.29	54.88	2.53	54.82	3.30	55.89	2.92	54.33	2.97
IN1	53.59	2.74	52.74	2.41	53.71	2.80	54.00	2.17	53.67	2.38	54.11	2.89	54.69	2.27	54.44	2.88	55.62	3.43	54.08	2.71
IN2	53.57	2.77	52.86	2.46	53.46	2.64	53.84	2.07	53.67	2.31	54.09	3.06	54.61	2.36	54.41	2.92	55.38	3.46	54.01	2.72
N1	53.19	2.72	52.35	2.39	53.19	2.63	53.58	2.33	53.14	2.40	53.72	2.75	54.28	2.45	53.93	2.71	54.78	3.20	53.60	2.65
N2	53.43	2.70	52.63	2.37	53.00	2.48	53.42	2.27	53.20	2.24	53.62	2.67	54.24	2.40	53.81	2.59	54.30	3.32	53.55	2.56
SN1	52.96	2.78	52.09	2.34	52.67	2.65	52.97	2.35	52.47	2.50	52.96	2.87	53.43	2.58	52.90	2.68	53.22	3.32	52.87	2.67
SN2	53.11	3.06	51.98	2.72	52.49	2.55	52.68	2.45	52.06	2.37	52.18	2.79	52.45	2.82	51.92	2.93	51.65	3.64	52.28	2.79

C: Center 2-mm, S1: Superior 2–5 mm, S2: Superior 5–6 mm, ST1: Superior Temporal 2–5 mm, ST2: Superior 5–6 mm, ST1: Temporal 2–5 mm, ST2: Temporal 5–6 mm, IT1: Inferior Temporal 2–5 mm, IT2: Inferior Temporal 5–6 mm, I1: Inferior 2–5 mm, I2, Inferior 5–6 mm, IN1: Inferior Nasal 2–5 mm, IN2: Inferior Nasal 5–6 mm,N1: Nasal 2–5 mm, N2: Nasal 5–6 mm, SN1: Superior Nasal 2–5 mm, SN2: Superior Nasal 5–6 mm, SD: Standard Deviation.

**Table 5:** Corneal epithelium thickness in the central (2-mm diameter), para-central (2 to 5-mm diameter), and peripheral (5 to 6-mm diameter), according to gender in 614 normal Chinese children.

Gender						
	Male		Female		Total	
	Mean	SD	Mean	SD	Mean	SD
	n = 312		n = 302		n = 614	
C	53.93	2.77	53.23	2.86	53.58	2.84
S1	52.63	2.69	51.68	2.63	52.16	2.70
S2	51.13	3.02	50.45	3.01	50.80	3.03
ST1	52.71	2.78	51.51	2.65	52.12	2.78
ST2	51.93	2.64	51.00	2.77	51.47	2.74
T1	52.91	2.84	51.93	2.91	52.43	2.91
T2	52.54	2.72	51.58	2.84	52.07	2.82
IT1	53.72	2.96	53.08	2.90	53.41	2.94
IT2	53.68	2.94	53.20	3.01	53.44	2.98
I1	54.36	2.95	53.93	2.82	54.15	2.90
I2	54.45	3.00	54.21	2.93	54.33	2.97
IN1	54.40	2.68	53.74	2.70	54.08	2.71
IN2	54.34	2.70	53.67	2.71	54.01	2.72
N1	54.04	2.57	53.15	2.67	53.60	2.65
N2	53.95	2.51	53.13	2.54	53.55	2.56
SN1	53.35	2.64	52.39	2.63	52.87	2.67
SN2	52.68	2.71	51.87	2.82	52.28	2.79

C: Center 2-mm, S1: Superior 2–5 mm, S2: Superior 5–6 mm, ST1: Superior Temporal 2–5 mm, ST2: Superior 5–6 mm, ST1: Temporal 2–5 mm, ST2: Temporal 5–6 mm, IT1: Inferior Temporal 2–5 mm, IT2: Inferior Temporal 5–6 mm, I1: Inferior 2–5 mm, I2, Inferior 5–6 mm, IN1: Inferior Nasal 2–5 mm, IN2: Inferior Nasal 5–6 mm,N1: Nasal 2–5 mm, N2: Nasal 5–6 mm, SN1: Superior Nasal 2–5 mm, SN2: Superior Nasal 5–6 mm, SD: Standard Deviation.

**Table 6:** Corneal epithelium thickness in the central (2-mm diameter), para-central (2 to 5-mm diameter), and peripheral (5 to 6-mm diameter), according to the quartile corneal curvature radius in normal Chinese children (n = 370).

Corneal Curvature Radius (mm)										
	7.15–7.65		7.66–7.83		7.84–8.00		8.01–8.60		Total	
	Mean	SD	Mean	SD	Mean	SD	Mean	SD	Mean	SD
	n = 88		n = 94		n = 91		n = 97		n = 370	
C	52.72	2.76	53.16	2.68	53.60	2.68	53.51	2.61	53.25	2.69
S1	50.78	2.39	51.90	2.81	51.95	2.59	52.36	2.46	51.77	2.63
S2	49.28	2.80	50.52	2.98	50.54	2.86	51.15	2.62	50.40	2.88
ST1	51.00	2.38	51.78	2.81	51.98	2.72	52.27	2.58	51.77	2.66
ST2	50.18	2.47	51.11	2.89	51.38	2.55	51.71	2.40	51.11	2.64
T1	51.50	2.60	51.98	2.89	52.37	2.76	52.55	2.75	52.11	2.77
T2	51.11	2.58	51.73	2.96	51.92	2.79	52.42	2.55	51.81	2.75
IT1	52.51	2.81	53.05	2.70	53.48	3.03	53.49	2.68	53.15	2.82
IT2	52.66	3.08	53.06	3.02	53.56	3.06	53.72	2.59	53.26	2.96
I1	53.32	2.88	53.88	2.87	54.38	2.91	54.18	2.65	53.95	2.84
I2	53.45	3.07	54.21	2.89	54.60	3.00	54.60	2.69	54.23	2.93
IN1	53.01	2.74	53.79	2.80	54.33	2.68	54.23	2.54	53.85	2.73
IN2	52.86	2.67	53.80	2.99	54.25	2.50	54.20	2.69	53.79	2.77
N1	52.47	2.58	53.27	2.73	53.67	2.57	53.76	2.48	53.31	2.63
N2	52.30	2.38	53.33	2.81	53.54	2.40	53.90	2.48	53.28	2.58
SN1	51.64	2.34	52.60	2.75	52.81	2.61	53.06	2.44	52.54	2.59
SN2	50.68	2.53	52.16	2.96	52.20	2.58	52.61	2.54	51.94	2.74

C: Center 2-mm, S1: Superior 2–5 mm, S2: Superior 5–6 mm, ST1: Superior Temporal 2–5 mm, ST2: Superior 5–6 mm, ST1: Temporal 2–5 mm, ST2: Temporal 5–6 mm, IT1: Inferior Temporal 2–5 mm, IT2: Inferior Temporal 5–6 mm, I1: Inferior 2–5 mm, I2, Inferior 5–6 mm, IN1: Inferior Nasal 2–5 mm, IN2: Inferior Nasal 5–6 mm, N1: Nasal 2–5 mm, N2: Nasal 5–6 mm, SN1: Superior Nasal 2–5 mm, SN2: Superior Nasal 5–6 mm, SD: Standard Deviation.
