# Supplementary material for: Corneal Epithelium Thickness Profile in 614 Normal Chinese Children Aged 7–15 Years Old
Source: Sci Rep. 2016 Mar 23;6:23482. doi: 10.1038/srep23482 (PMC4804327; doi:10.1038/srep23482)
Supplement: Supplementary Information [file srep23482-s1.doc]

Title: Corneal Epithelium Thickness Profile in 614 Normal Chinese Children Aged 7-15 Years Old

Authors: Yingyan Ma, Xiangui He, Xiaofeng, Lina Lu, Jianfeng Zhu, Haidong Zou

Supplementary Table 1. The minimum, maximum, difference between the minimum and maximum corneal epithelium thickness, and the standard deviation measured by Fourier-domain optical coherence tomography reported in previous literatures.

|  | **N (subjects)** | **Age (Mean ±SD)** | **Min (µm)** | **Max (µm)** | **SD** | **Min-Max** |
| --- | --- | --- | --- | --- | --- | --- |
| **Yang et al^26^** | 36 | 67.47 ± 5.40 | 46.7 ± 3.2 | 56.3 ±4.2 | 2.2 ±1.1 | -9.6 ± 4.9 |
| **Li et al^4^** | 145 | 47.6 ± 13.9 | 46.0 ± 4.3 | / | / | -8.8 ± 3.5 |
| **Kanellopoulos et al^21^** | 35 | 47.5 ± 15.6 | 48.4 | 56.3 | 1.8 | / |
| **Cui et al^22^** | 35 | 43. 57 ± 17.36 | 47.91± 5.9 | 56.60 ± 3.88 | 2.04 ± 1.69 | -8.57 ± 6.57 |
| **Yang et al^26^** | 45 | 14.29 ± 3.66 | 49.9 ±2.7 | 56.2 ± 3.0 | 1.5 ± 0.5 | -6.4 ± 2.1 |
| **Present study** | 614 | 11.24 ±2.34 | 50.01 ± 2.84 | 56.63 ±3.20 | 1.37 ±0.58 | -6.01 ± 2.72 |
|  |  |  |  |  |  |  |
| Min, minimum corneal epithelium thickness  Max, maximum corneal epithelium thickness  Min-Max, differences of minimum corneal epithelium thickness and maximum corneal epithelium thickness  SD, standard deviation | | | | | | |
